# Immune checkpoint blockade PD-1 therapy for primary liver cancer: incidence and influencing factors of thyroid dysfunction

**DOI:** 10.1186/s13027-022-00476-6

**Published:** 2022-12-29

**Authors:** Huili Wu, Fang Xiong, Xuli Bao, Jun Lu

**Affiliations:** Department of Oncology, Beijing YouAn Hospital, Capital Medical University, 8 Xitoutiao, Youwai Street, Beijing, 100069 China

**Keywords:** Primary liver cancer, Immune checkpoint inhibitors, Thyroid function, Immune related adverse reactions

## Abstract

**Objectives:**

To investigate the incidence and influencing factors of thyroid dysfunction (TD) in patients with primary liver cancer (PLC) induced by PD-1 monoclonal antibodies.

**Methods:**

Clinical data were collected from 195 PLC patients treated with PD-1. They were divided into TD group and normal thyroid function (NTF) group, and further divided into TD subgroups, the differences between groups and subgroups were analyzed.

**Results:**

A total of 113 of 195 (57.9%) PLC patients developed TD. The positive rate of thyroid antibody (20.6% vs. 0%, *P* = 0.041) and the median value of TSH (6.20 vs. 2.16 mU/L, *P* = 0.000) in TD group were higher than those in NTF group. Ten patients (8.8%) had the CTCAE grade of TD above grade 3, of which 2 patients died of liver failure. There were 20 patients (17.7%) in hyperthyroidism group and 93 patients (82.3%) in hypothyroidism group. The decompensated cirrhosis in hyperthyroidism group was lower than that in hypothyroidism group (33.3% vs. 65.6%, *P* = 0.010), and the proportion of patients who had previously received surgical treatment was higher than that in hypothyroidism group (35.0% vs. 9.7%, *P* = 0.003); The proportion of clinical hyperthyroidism was higher than that of clinical hypothyroidism (70.0% vs. 31.2%, *P* = 0.001), the proportion of decompensated liver cirrhosis in clinical hyperthyroidism group was lower than that in clinical hypothyroidism group (23.1% vs. 68.0%, *P* = 0.022), and the proportion of previous or combined surgical resection was much higher than that in clinical hypothyroidism group (42.9% vs. 7.1%, *P* = 0.018); The proportion of decompensated cirrhosis in primary TD group was lower than that in secondary TD group (36.5% vs. 83.3%, *P* = 0.002), and the proportion of patients using antitumor targeted drugs was higher than that in secondary TD group (73.1% vs. 45.0%, *P* = 0.014).

**Conclusion:**

Patients with PLC had high incidence of TD after receiving PD-1 treatment, primary or subclinical hypothyroidism was the main manifestation type, which was related to the degree of disease and treatment.

## Introduction

Primary liver cancer (PLC) is a kind of malignant tumor originating from hepatocytes or intrahepatic bile duct epithelial cells. It is characterized by concealed onset, high degree of malignancy, rapid progress and difficult treatment. The incidence rate and mortality of PLC in China ranked first in the world, with a 5-year survival of only 12.1% [1]. At present, PD-1/PD-L1 antibody as checkpoint inhibitor combined with antitumor targeted drugs had become the first-line treatment of unresectable PLC. Previous clinical studies had shown that PD-1 was generally safe and well tolerated in the treatment of tumors. However, as a new generation of immunotherapeutic drugs, PD-1 inhibitors reactivate the suppressed anti-tumor immune function of the body. Therefore, it is significantly different from traditional anti-tumor drugs, that is, the damage of PD-1 to the immune system is a unique adverse reaction. Endocrine organs, especially thyroid, are the most common target organs of immune-mediated adverse reactions. Previous studies had also observed that PD-1 had different degrees of thyroid dysfunction in the treatment of different tumors [2], but so far, there was few real-world studies on the occurrence of thyroid dysfunction in PLC patients after treatment, especially lack of detailed research on the manifestation types, clinical characteristics and influencing factors related to thyroid dysfunction. Therefore, this study retrospectively analyzed the clinical data of a large number of PLC patients treated with PD-1in our center, in order to provide more clinical experience for the prevention, diagnosis, treatment and management of TD adverse reactions after immunotherapy.

## Patients and methods

### Patients

**Fig. 1:**
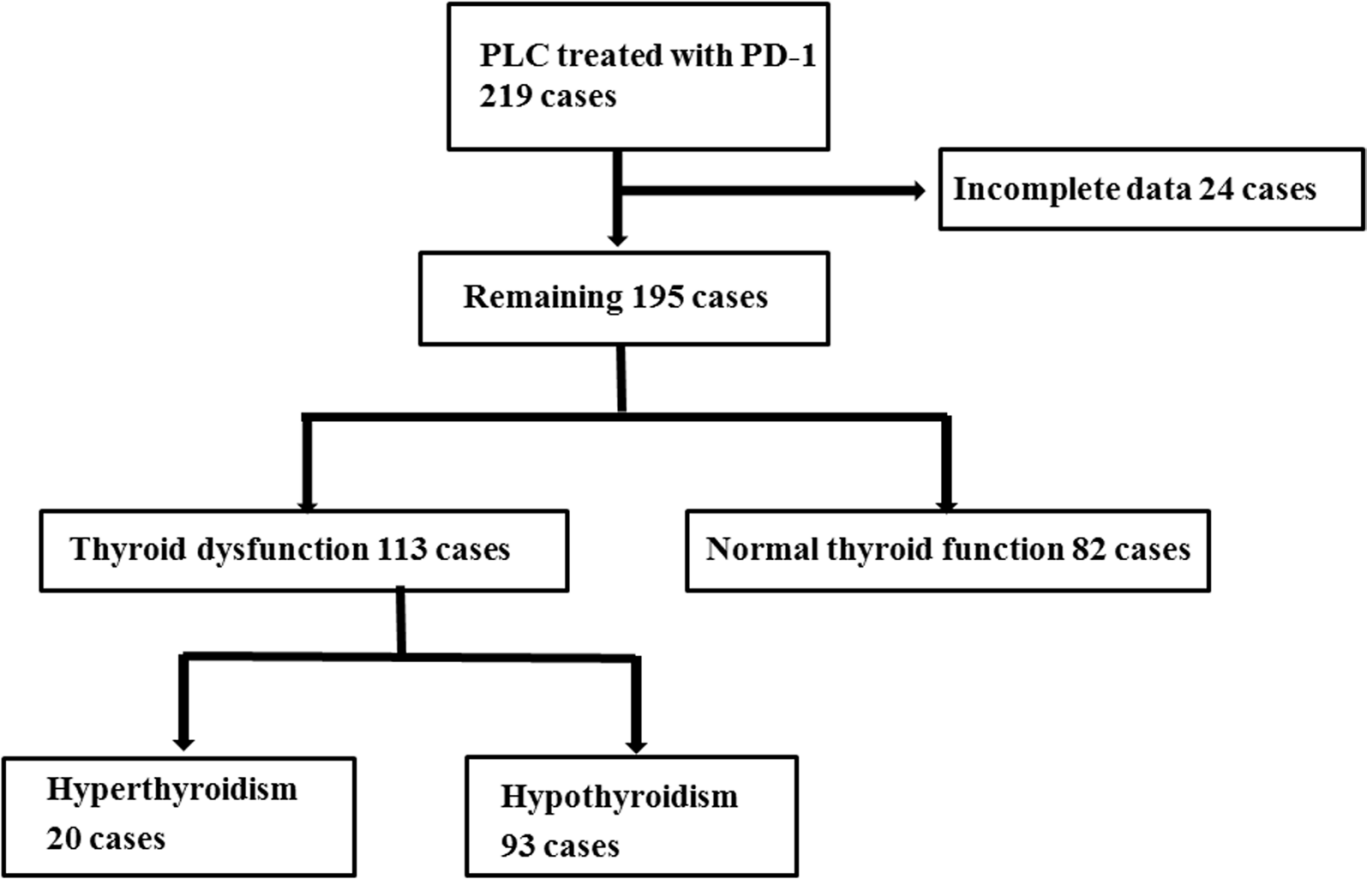
Flow chart of case selection

We performed a retrospective study of the medical records of all 219 PLC patients who underwent PD-1(Camrelizumab or Sintilimab) therapy between January 2020 and September 2021. The diagnosis standard of PLC refers to the staging standard of BCLC strategy 2022 update [3]. Inclusion criteria: (1) PLC was diagnosed by clinical or histopathological examination; (2) received at least 2 cycles of standardized PD-1 treatment; (3) received analyzable clinical medical records or follow-up data. Exclusion criteria: (1) with thyroid disease history, operation history and examination determined before PD-1 treatment have TD, such as abnormal thyroid stimulating hormone (TSH), free thyroxine 4(FT4) or free thyroxine 3(FT3); (2) received thyroid regional radiotherapy; (3) taking drugs that affect thyroid function; (4) other immune checkpoint inhibitors were used before treatment; (5) incomplete information. After screening according to above criteria, a total of 24 cases were excluded, and the remaining 195 cases were included in the retrospective analysis (see Fig. 1).

### Data collection

The basic information, clinical data and prognosis of all patients included in the group were collected by querying the electronic medical record system or the attending physician, as well as telephone follow-up of patients and their families. The collection contents include: gender, age, body mass index(BMI), diabetes, hepatitis B /C virus infection, liver cirrhosis diagnosis, tumor classification and stage, history of tumor treatment and starting time, thyroid function and detection time before and after treatment, thyroid autoantibody detection, etc.

### Laboratory examination

TSH, FT3 and FT4 were detected by immunoluminescence method (Abbott architect i2000sr automatic immunoanalyzer), and thyroglobulin antibody (TG-Ab), thyroid peroxidase antibody (TPO-Ab) and thyrotropin receptor antibody (TSHR-Ab) were detected by automatic Electrochemiluminescence Method (Roche Cobase 801 chemiluminescence instrument). The normal value range was set as follows: TSH:0.35–4.94 mU/L, FT3:2.90–4.90 pmol/L, TT3:0.99–2.34 pmol/L, FT4:9.01–19.05 pmol/L, TT4:62.68–150.84 pmol/L.

### Evaluation of thyroid function

For the diagnostic criteria of immunotherapy related hyperthyroidism and hypothyroidism, referred to 2021 NCCN Guidelines Insights: Management of Immunotherapy-Related Toxicities [4] and 2016 American Thyroid Association Guidelines for Diagnosis and Management of Hyperthyroidism and Other Causes of Thyrotoxicosis [5]. They were divided into four groups according to symptoms: (1) subclinical hypothyroidism group: TSH increased, FT4/FT3 was normal, and there were no clinical symptoms; (2) clinical hypothyroidism: TSH increased > 10 mU/L, FT4/FT3 decreased, with clinical symptoms; (3) subclinical hyperthyroidism: TSH decreased, FT4/FT3 normal, no clinical symptoms; (4) clinical hyperthyroidism: TSH decreased and FT4/FT3 increased, with obvious clinical symptoms. According to the etiology: (1) primary TD: elevated TSH with decreased FT4/FT3, or decreased TSH with increased FT4/FT3; (2) secondary TD: TSH is normal and FT4/FT3 decreases or increases. Immune related adverse reactions (irAEs) referred to the standard of adverse event reporting terminology of American Cancer Center(CTCAE5.0) [6], which was divided into five levels according to the severity: Grade 1: Mild, asymptomatic or mild, only clinical or diagnostic findings, without treatment; Grade 2: moderate, requiring minor, local or non-invasive treatment, and age-related instrumental activities of daily living are limited; Grade 3: Serious or medically significant but not immediately life-threatening, resulting in hospitalization or prolonged hospitalization, disability, and limited self-care activities of daily living; Grade 4: life threatening, requiring emergency treatment; Grade 5: death related to adverse reactions.

### Treatment and follow-up

Patients needed at least one cycle of PD-1 treatment. Refer to the drug instructions for the dosage. The follow-up time was at least two cycles after receiving PD-1 treatment or 3 months after stopping PD-1 treatment.

### Statistical analysis

SPSS 16.0 and GraphPad Prism 5.02 were used for statistical analysis. The normal distribution of measurement data was expressed as mean ± standard deviation. T-test or Kruskal–Wallis ANOVA test was used for comparison among groups. Skewness distribution of measurement data was expressed as medians and ranges, Mann–Whitney U test was used for comparison among groups. The counting data were expressed by the number of cases and percentages, and the comparisons between groups were performed by *χ*^2^ test or Fisher’s exact probability method. The difference was statistically significant (*P* < 0.05).

## Result

### Incidence and clinical characteristics of TD

After excluding 24 patients with incomplete data, 195 of 219 PLC patients were included in the analysis, including 154 males (79.0%) and 41 females (21.0%), with an average age of 58.1 ± 10.2 years. All patients had tested for normal thyroid function before treatment, including 182 patients with hepatocellular carcinoma, accounting for 93.3%, and the other 13 patients were intrahepatic cholangiocarcinoma and hepatic adenocarcinoma. There were 113 cases (57.9%) of TD and 82 cases (42.1%) of NTF after treatment (Fig. 1). There was no significant difference between TD group and NTF group in gender, age, BMI, diabetes, evaluation time, tumor classification and stage, combined virus infection, combined liver cirrhosis and previous treatment methods, but the positive rate of thyroid antibody in TD group was significantly higher than that in NTF group (20.6% vs. 0%, *P* = 0.041). There was no significant difference in thyroid function between the two groups before treatment, while the TSH level in TD group was higher than that in NTF group (6.20 vs. 2.16, *P* = 0.000), and there was significant statistical difference (Table 1). The treatment of patients with TD conforms to Management of Immune-Related Adverse Events in Patients Treated With Immune Checkpoint Inhibitor Therapy: ASCO Guideline Update [7].

**Table 1:** Comparison of clinical data between thyroid dysfunction group and normal thyroid function group

	Thyroid dysfunction group (n = 113)	Normal thyroid function group (n = 82)	*P*	*t/ x*^*2*^*/Z*
Gender (n, %)			0.131	2.280
Male	85(75.2)	69(84.1)		
Female	28(24.8)	13(15.9)		
Age(years,*x* ± *s*)	58.6 ± 10.7	57.3 ± 9.6	0.380	0.880
BMI	23.5 ± 3.3	22.8 ± 4.1	0.235	1.193
Diabetes			0.877	0.024
Yes	17(15.0)	13(15.9)		
No	96(85.0)	69(84.1)		
Evaluation time [weeks,*M*(*Q1,Q3*)]	10.0(3.0, 25.5)	10.0(5.7, 21.0)	0.503	0.669
Hepatocellular carcinoma (n,%)			0.786	0.074
Yes	105(92.9)	77(93.9)		
No	8(7.1)	5(6.1)		
Staging of BCLC system(n,%)			0.815	0.054
B	25(22.1)	17(20.7)		
C	88(77.9)	65(79.3)		
HBV or HCV(n,%)	101(85.8)	69(79.3)	0.280	1.165
Cirrhosis (n,%)	92(80.5)	68(87.8)	0.786	0.074
Decompensation stage (n,%)			0.371	0.799
Yes	50(44.2)	39(47.6)		
No	42(55.8)	43(52.4)		
Previous or combined treatment (n,%)				
Surgical resection	16(14.2)	16(19.5)	0.319	0.993
TAE or TACE	85(75.2)	52(63.4)	0.094	2.810
Tumor ablation	55(48.7)	42(51.2)	0.725	0.123
Targeted agent	77(68.1)	55(67.1)	0.875	0.025
Systemic chemotherapy	6(5.3)	4(4.9)	1.000	0.000
Radiotherapy	14(12.4)	5(6.1)	0.144	2.139
Name of PD-1 monoclonal antibody (n,%)			0.591	0.289
Sintilimab	59(52.2)	46(56.1)		
Camrelizumab	54(47.8)	36(43.9)		
Thyroid antibody (n,%)			**0.041**	–
Positive	7(20.6)	0(0.0)		
Negative	27(79.4)	19(100.0)		
Thyroid function before treatment of PD-1				
TSH[mU/L,*M*(*Q1,Q3*)]	2.23(1.42, 3.88)	2.05(1.33, 3.45)	0.620	0.495
FT4[pmol/L,*M*(*Q1,Q3*)]	13.25(11.84, 15.00)	12.59(11.37, 14.83)	0.223	1.219
FT3[pmol/L,*M*(*Q1,Q3*)]	3.86(3.18, 4.42)	3.80(3.27, 4.32)	0.962	0.047
Thyroid function after treatment of PD-1				
TSH[mU/L,*M*(*Q1,Q3*)]	6.20(1.42, 10.17)	2.16(1.52, 3.22)	**0.000**	5.198
FT4[pmol/L,*M*(*Q1,Q3*)]	12.18(10.54, 14.33)	12.32(11.60, 13.89)	0.398	0.845
FT3[pmol/L,*M*(*Q1,Q3*)]	3.68(2.93, 4.40)	3.80(2.96, 4.28)	0.675	0.420

BCLC:Barcelona Clinic Liver Cancer; TAE: transarterial embolization;TACE: transar-terial chemoembolization; PD-1: programmed cell death protein 1; TSH: abnormal thyroid stimulating hormone; FT4: free thyroxine 4; FT3: free thyroxine 3

### Classification and clinical characteristics of TD

According to the thyroid test results, 113 patients with TD were divided into hyperthyroidism group including 20 patients (17.7%) and hypothyroidism group including 93 patients (82.3%), and 93 patients with hypothyroidism (82.3%).In 195 treated patients, the total incidence of hyperthyroidism was 10.3% and hypothyroidism was 47.6%; According to the etiology, 113 patients with TD were divided into primary group including 93 patients (82.3%) and secondary group including 20 patients (17.7%); According to the symptoms, 113 patients with TD were divided into clinical group including 42 patients(37.2%) and subclinical group including 71 patients with subclinical group (62.8%); According to the severity of adverse reactions, patients were divided into grade 1–5 and 113 patients were in grade 1–3, of which grade 3 accounted for only 8.8%. In addition, in the hyperthyroidism group, clinical hyperthyroidism and subclinical hyperthyroidism accounted for 70% and 30.0% respectively, while in the hypothyroidism group, clinical hypothyroidism accounted for 31.2% and subclinical hypothyroidism 68.8%, showing significant statistical difference (*P* = 0.001) (Table 2).

**Table 2:** Clinical classification of thyroid dysfunction related to PD-1 monoclonal antibody therapy in primary liver cancer

	n	Hyperthyroidism (n = 20)	Hypothyroidism (n = 93)	*P*	*t/x*^*2*^*/Z*
Classification by causes (n,%)					
Primary thyroid dysfunction	93	19(95.0)	74(79.6)	0.188	1.735
Secondary thyroid dysfunction	20	1(5.0)	19(20.4)		
Classification by symptom (n,%)					
Clinical thyroid dysfunction	42	14(70.0)	28(31.2)	**0.001**	11.217
Subclinical thyroid dysfunction	71	6(30.0)	65(68.8)		
Classification by adverse reaction severity (n,%)				0.328	2.230
Grade 1	61	8(40.0)	53(57.0)		
Grade 2	42	9(45.0)	33(35.5)		
Grade 3	10	3(15.0)	7(7.5)		
Grade 4	0	0(0.0)	0(0.0)		
Grade 5	0	0(0.0)	0(0.0)		

### Analysis of clinical characteristics of TD subgroup

The proportion of decompensated cirrhosis in hypothyroidism group was significantly higher than that in hyperthyroidism group (65.6% vs. 33.3%, *P* = 0.010), while the proportion of previous surgery in hyperthyroidism group was higher than that in hypothyroidism group (35.0% vs. 9.7%, *P* = 0.003); There was no difference between the two groups in combination with other treatments, such as intervention, ablation, targeted drugs, chemotherapy and radiotherapy, and there was no significant difference between the two groups in gender, age, BMI, diabetes, evaluation time, tumor classification and stage, combined virus infection and thyroid antibody expression (all *P* > 0.05) (Table 3).

**Table 3:** Clinical characteristics of thyroid dysfunction related to PD-1 monoclonal antibody therapy in primary liver cancer

	Hyperthyroidism (n = 20)	Hypothyroidism (n = 93)	*P*	*t/ x*^*2*^*/Z*
Gender (n, %)			0.460	1.057
Male	17(85.0)	69(74.2)		
Female	3(15.0)	24(25.8)		
Age(years,*x* ± *s*)	56.5 ± 8.1	58.9 ± 11.1	0.353	0.933
BMI	23.7 ± 2.9	23.3 ± 3.6	0.729	0.348
Diabetes			1.000	0.000
Yes	3(15.0)	15(16.1)		
No	17(85.0)	78(83.9)		
Evaluation time [weeks,*M*(*Q1,Q3*)]	14.0(9.0, 29.5)	11.0(3.0, 25.5)	0.117	1.566
Hepatocellular carcinoma (n,%)			0.347	-
Yes	20(100.0)	85(91.4)		
No	0(0.0)	8(8.6)		
Staging of BCLC system(n,%)			1.000	0.000
B	4(15.0)	21(19.4)		
C	16(85.5)	72(80.6)		
HBV or HCV(n,%)	19(77.4)	83(85.0)	0.710	0.138
Cirrhosis (n,%)	18(80.5)	65(87.8)	0.384	0.759
Decompensation stage (n,%)	6(33.3)	61(65.6)	**0.010**	6.559
Yes	12(66.7)	32(34.4)		
No				
Previous or combined treatment (n,%)	7(35.0)	9(9.7)	**0.003**	8.684
Surgical resection	16(80.0)	69(74.2)	0.585	0.298
TAE or TACE	11(55.0)	44(47.3)	0.533	1.389
Tumor ablation	13(85.0)	64(68.8)	0.740	0.110
Targeted agent	0(0.0)	6(6.5)	0.588	-
Systemic chemotherapy	1(5.0)	13(14.0)	0.464	0.535
Radiotherapy	0(0.0)	13(14.0)	0.120	-
Name of PD-1 monoclonal antibody (n,%)			0.827	0.048
Sintilimab	10(50.0)	49(52.7)		
Camrelizumab	10(50.0)	44(47.3)		
Thyroid antibody (n,%)			1.000	0.000
Positive	2(22.2)	5(20.0)		
Negative	7(77.9)	20(80.0)		

BCLC:Barcelona Clinic Liver Cancer; TAE: transarterial embolization;TACE: transar-terial chemoembolization; PD-1: programmed cell death protein 1; TSH: abnormal thyroid stimulating hormone; FT4: free thyroxine 4; FT3: free thyroxine 3

The proportion of patients with decompensated liver cirrhosis in primary TD group was significantly lower than that in secondary TD group (36.5% vs. 83.3%, *P* = 0.002), and the proportion of patients treated with anti-cancer targeted drugs in primary TD group was higher than that in secondary thyroid dysfunction group (73.1% vs. 45.0%, *P* = 0.014). In addition, the positive rate of thyroid antibody in primary TD group was 31.8%, while there was no positive thyroid antibody in secondary abnormal thyroid function group. The difference between the two groups was statistically significant (*P* = 0.036) (Table 4).

**Table 4:** clinical characteristics of thyroid dysfunction related to PD-1 monoclonal antibody therapy in primary liver cancer

	Primary thyroid dysfunction (n = 93)	Secondary thyroid dysfunction (n = 20)	*P*	*t/ x*^*2*^*/Z*
Gender (n, %)			0.264	1.247
Male	68(73.1)	17(85.0)		
Female	25(26.9)	3(15.0)		
Age(years,*x* ± *s*)	58.0 ± 10.7	61.6 ± 9.9	0.174	1.368
BMI	22.1 ± 2.7	23.6 ± 3.5	0.132	1.520
Diabetes			1.000	0.000
Yes	15(16.1)	3(15.0)		
No	78(83.9)	17(85.0)		
Evaluation time [weeks,*M*(*Q1,Q3*)]	12.0(3.0, 27.8)	10.0(3.3, 17.8)	0.412	0.821
Hepatocellular carcinoma (n,%)			0.936	0.007
Yes	87(93.5)	18(90.0)		
No	6(6.5)	2(10.0)		
Staging of BCLC system(n,%)			0.350	0.875
B	19(20.4)	6(30.0)		
C	74(76.0)	14(70.0)		
HBV or HCV(n,%)	83(77.4)	19(85.0)	0.710	0.138
Cirrhosis (n,%)	74(80.5)	18(87.8)	0.441	0.595
Decompensation stage (n,%)	27(36.5)	15(83.3)	**0.002**	9.166
Yes	41(63.5)	3(16.7)		
No				
Previous or combined treatment (n,%)	15(16.1)	1(5.0)	0.346	0.887
Surgical resection	69(74.2)	16(80.0)	0.795	0.086
TAE or TACE	43(46.2)	12(60.0)	0.264	1.248
Tumor ablation	68(73.1)	9(45.0)	**0.014**	5.995
Targeted agent	5(5.4)	1(5.0)	1.000	0.000
Systemic chemotherapy	12(12.9)	2(10.0)	1.000	0.000
Radiotherapy	12(12.9)	1(5.0)	0.536	0.383
Name of PD-1 monoclonal antibody (n,%)			0.319	0.992
Sintilimab	50(53.8)	9(45.0)		
Camrelizumab	43(46.2)	11(55.0)		
Thyroid antibody (n,%)			**0.036**	-
Positive	7(31.8)	0(0.0)		
Negative	15(68.2)	12(100.0)		

BCLC:Barcelona Clinic Liver Cancer; TAE: transarterial embolization;TACE: transar-terial chemoembolization; PD-1: programmed cell death protein 1; TSH: abnormal thyroid stimulating hormone; FT4: free thyroxine 4; FT3: free thyroxine 3

There were no significant abnormalities between subclinical TD group and clinical TD group in terms of gender, age, diabetes, evaluation time, tumor classification and stage, combined virus infection, combined liver cirrhosis, previous treatment, immunosuppressant types and thyroid antibody expression (all *P* > 0.05) except BMI(*P* = 0.032) (Table 5). However, the proportion of decompensated cirrhosis in the clinical hyperthyroidism group was significantly lower than that in the clinical hypothyroidism group (23.1% vs. 68.0%, *P* = 0.022), but the previous or combined surgical resection in the clinical hyperthyroidism group was much higher than that in the clinical hypothyroidism group (42.9% vs. 7.1%, *P* = 0.018) (Table 6).

**Table 5:** Clinical characteristics of thyroid dysfunction associated with PD-1 monoclonal antibody therapy in primary liver cancer

	Clinical thyroid dysfunction (n = 71)	Subclinical thyroid dysfunction (n = 42)	*P*	*t/ x*^*2*^*/Z*
Gender (n, %)			0.854	0.034
Male	53(73.1)	32(85.0)		
Female	18(26.9)	10(15.0)		
Age(years,*x* ± *s*)	57.6 ± 11.0	60.1 ± 9.6	0.219	1.235
BMI	24.5 ± 3.7	22.8 ± 3.1	0.032	2.18
Diabetes			0.144	2.132
Yes	8(11.3)	9(21.4)		
No	63(88.7)	33(78.6)		
Evaluation time [weeks,*M*(*Q1,Q3*)]	10.0(3.0, 25.5)	10.0(5.7, 21.0)	0.503	0.669
Hepatocellular carcinoma (n,%)			0.719	0.546
Yes	65(92.9)	40(91.5)		
No	6(7.1)	2(6.1)		
Staging of BCLC system(n,%)			0.4480	0.499
B	17(24.3)	8(19.0)		
C	53(75.7)	35(81.0)		
HBV or HCV(n,%)	63(88.7)	38(90.5)	1.000	0.000
Cirrhosis (n,%)	54(80.5)	38(87.8)	0.098	2.736
Decompensation stage (n,%)	30(55.6)	20(52.6)	0.782	0.077
Yes	24(44.4)	18(47.4)		
No				
Previous or combined treatment (n,%)	7(9.9)	9(21.4)	0.088	2.906
Surgical resection	51(71.8)	34(81.0)	0.278	1.178
TAE or TACE	33(46.5)	22(52.4)	0.544	0.368
Tumor ablation	49(69.0)	28(66.7)	0.796	0.067
Targeted agent	4(5.6)	2(4.8)	1.000	0.000
Systemic chemotherapy	10(14.1)	4(9.5)	0.678	0.173
Radiotherapy	9(12.7)	4(9.5)	0.840	0.041
Name of PD-1 monoclonal antibody (n,%)			0.254	1.303
Sintilimab	40(56.3)	19(45.2)		
Camrelizumab	31(44.4)	23(54.8)		
Thyroid antibody (n,%)			0.306	1.050
Positive	2(11.1)	5(31.3)		
Negative	16(88.9)	11(68.8)		

BCLC:Barcelona Clinic Liver Cancer; TAE: transarterial embolization;TACE: transar-terial chemoembolization; PD-1: programmed cell death protein 1; TSH: abnormal thyroid stimulating hormone; FT4: free thyroxine 4; FT3: free thyroxine 3

**Table 6:** Clinical characteristics of thyroid dysfunction associated with PD-1 monoclonal antibody therapy in primary liver cancer

	Clinical hyperthyroidism (n = 14)	Clinical hypothyroidism (n = 28)	*P*	*t/ x*^*2*^*/Z*
Gender (n, %)			0.522	0.410
Male	12(85.7)	20(71.4)		
Female	2(14.3)	8(28.6)		
Age(years,*x* ± *s*)	61.5 ± 9.9	57.1 ± 8.7	0.167	1.408
BMI	24.9 ± 2.6	24.3 ± 3.9	0.698	0.394
Diabetes			1.000	0.000
Yes	3(21.4)	5(17.9)		
No	11(78.6)	23(82.1)		
Evaluation time [weeks,*M*(*Q1,Q3*)]	11.5(3.0, 30.0)	11.0(3.0, 23.3)	0.742	0.348
Hepatocellular carcinoma (n,%)			0.545	-
Yes	14(100.0)	26(92.9)		
No	0(0.0)	2(7.1)		
Staging of BCLC system(n,%)			1.000	0.000
B	3(15.0)	5(19.4)		
C	13(85.5)	23(80.6)		
HBV or HCV(n,%)	14(77.4)	24(85.0)	0.283	-
Cirrhosis (n,%)	13(80.5)	25(87.8)	1.000	0.000
Decompensation stage (n,%)	3(23.1)	17(68.0)	**0.022**	5.238
Yes	10(76.9)	8(32.0)		
No				
Previous or combined treatment (n,%)	6(42.9)	2(7.1)	**0.018**	5.527
Surgical resection	12(85.7)	22(78.6)	0.578	0.309
TAE or TACE	8(57.1)	14(50.0)	0.662	0.191
Tumor ablation	9(64.3)	19(67.9)	0.817	0.05
Targeted agent	0(0.0)	2(7.1)	0.545	-
Systemic chemotherapy	1(7.1)	3(10.7)	1.000	0.000
Radiotherapy	0(0.0)	4(14.3)	0.283	-
Name of PD-1 monoclonal antibody (n,%)			0.273	1.201
Sintilimab	8(57.1)	11(39.3)		
Camrelizumab	6(42.9)	17(60.7)		
Thyroid antibody (n,%)			1.000	0.000
Positive	2(40.0)	3(27.3)		
Negative	3(60.0)	8(72.7)		

BCLC:Barcelona Clinic Liver Cancer; TAE: transarterial embolization;TACE: transar-terial chemoembolization; PD-1: programmed cell death protein 1; TSH: abnormal thyroid stimulating hormone; FT4: free thyroxine 4; FT3: free thyroxine 3

Grade 3 adverse events occurred in 10 patients with TD. The clinical manifestations, characteristics and prognosis were shown in Tables 7 and 8. Among the 10 patients, there were 7 males and 3 females, aged between 43 and 84 years; Nine patients had HBV infection and cirrhosis, of which 3 patients were decompensated cirrhosis; Eight patients with liver cancer stage were stage C, two patients were stage B, nine patients had other treatment schemes, and two patients had thyroid antibody positive. The earliest time for patients to find TD was 1 week after treatment, and the latest was 36 weeks; There were 3 cases of hyperthyroidism, the main symptoms were palpitation, emaciation, fatigue and irritability, and 7 cases of hypothyroidism, the main symptoms were loss of appetite, fatigue, edema and fatigue; Among the 10 patients, only one patient recovered thyroid function after treatment and did not interrupt PD-1 treatment, 4 patients interrupted PD-1 treatment but recovered thyroid function after treatment, 1 patient partially recovered thyroid function, 2 patients did not fully recover thyroid function and died during the period, and the direct cause of death was liver failure.

**Table 7:** The clinical characteristics of patients with thyroid dysfunction caused by PD-1 monoclonal antibody therapy classified as grade 3 by CTCAE

Case	Sex	Age	Name of PD-1	Hepatitis virus	Cirrhosis	Decompensation stage	Staging of BCLC system	Previous or combined treatment	Thyroid antibody
1	M	67	S*	HBV	Yes	Yes	C	TACE, Targeted agent	No detect
2	F	70	C**	No	No	–	C	None	Negative
3	M	43	C	HBV	Yes	Yes	C	TACE	Negative
4	F	49	C	HBV	No	–	C	TACE, Tumor ablation, Systemic chemotherapy	No detect
5	M	62	C	HBV	Yes	No	C	Surgical resection, TACE, Tumor ablation, Targeted agent, Radiotherapy	TPO, TG positive
6	F	58	C	HBV	Yes	No	C	TACE, Tumor ablationTargeted agent, Radiotherapy	No
7	M	58	C	HBV	Yes	No	C	TACE, Tumor ablation, Targeted agent	No
8	M	67	C	HBV	Yes	No	B	TACE, Tumor ablation, Targeted agent	No
9	M	47	C	HBV	Yes	No	B	Surgical resection, TACE, Tumor ablation	No
10	M	84	C	HBV	Yes	Yes	C	Targeted agent	TPO, TG positive

*Sintilimab; **Camrelizumab; TACE: transar-terial chemoembolization; PD-1: programmed cell death protein 1; TPO: thyroid peroxide-se antibody; TG: thyroglobulin antibody;

**Table 8:** The symptoms and prognosis of patients with thyroid dysfunction caused by PD-1 monoclonal antibody therapy classified as grade 3 by CTCAE

Case	Discovery time(week)	classification	First symptoms	Medication	PD-1 interrupt	Recovery of thyroid function	Tumor evaluation	Prognosis
1	8	Primary hyperthyroidism	Palpitation	Methimazole, propranolol	Yes	Full recovery	shrinkage	Persistence
2	12	Secondary hyperthyroidism	Loss of appetite, fatigue	Levothyroxine	Yes	Not recovered	progression	Death
3	1	Secondary hyperthyroidism	Loss of appetite, fatigue, edema	Levothyroxine	Yes	Not recovered	progression	Death
4	26	Primary hyperthyroidism	fatigue	Levothyroxine	No	Full recovery	progression	Persistence
5	28	Primary hyperthyroidism	Emaciation, fatigue	Methimazole	Yes	Partial recovery	Stability	Persistence
6	36	Primary hyperthyroidism	Loss of appetite, fatigue	Levothyroxine	Yes	Full recovery	Stability	Persistence
7	24	Primary hyperthyroidism	Edema	Levothyroxine	Yes	Full recovery	progression	Persistence
8	12	Primary hyperthyroidism	Edema, fatigue	Levothyroxine	Yes	Full recovery	Stability	Persistence
9	3	Primary hyperthyroidism	Fatigue, fidgety	unclear	Yes	Partial recovery	progression	Persistence
10	35	Secondary hyperthyroidism	Edema, fatigue	non-administration	Yes	unclear	progression	Persistence

## Discussion

With the widespread use of immunosuppressant in patients with PLC, we should not only pay attention to the curative effect, but also attach importance to the adverse reactions. TD is one of the common immune related adverse reactions caused by immunosuppressants. In this retrospective study, we found that more than half of PLC patients had TD after treatment of PD1, most of them showed hypothyroidism, mainly primary, subclinical and severity grade 1–2. The degree of liver cirrhosis and treatment methods may have an impact on TD. The vast majority of TD patients have a good prognosis.

At present, the reports of TD caused by immunosuppressant PD-1 monoclonal antibody treatment mostly come from clinical trials, rather than real-world studies, especially less studies of PD-1 monoclonal antibody treatment of PLC. As a real-world retrospective study, through the analysis of the clinical data of PLC patients after PD-1 treatment, we found that the incidence of thyroid adverse reactions can be as high as 57.9%, higher than 3.4–47.7% [2, 8], reported in previous Meta analysis of drug clinical trials, 42.37% reported by Wei Fenfen [9] and 29.0% reported by Yang Zizhong [10]. There were two main reasons for this difference. On the one hand, previous studies mostly involved lung cancer, melanoma, gastrointestinal tumors, breast cancer, hematological system tumors, etc., while PLC patients were rarely included in the above studies. PLC patients often had hepatitis B (C) virus infection and liver cirrhosis, accompanied by worse thyroid function. In our study, 80% of PLC patients were in Barcelona phase C and complicated with hepatitis B (C) virus infection and cirrhosis. On the other hand, less than 20% of PLC patients in our study have previously undergone surgical resection, and more than half of them have received TAE/TACE, tumor ablation and targeted drug therapy, which will promote the production of TD [11, 12]. In addition, the selection of therapeutic drugs may also affect the incidence of TD. There had few reports on the use of cindilimab and carrelizumab in previous studies, although there was no significant difference between the two antibodies in the incidence, type and severity of TD. At present, the pathogenesis of TD in PLC patients caused by PD-1 treatment is not clear, which may be related to patients' basic thyroid diseases, basic level of thyroid stimulating hormone and thyroid antibody, changes in the number and function of immune cells, tumor microenvironment, homology of antigen expressed between tumor and thyroid tissue, BMI, etc. [13, 14]. It is speculated that it may be the direct effect of drugs on thyroid function or the result secondary to thyroiditis. We also found that the BMI index of patients with clinical TD was higher than that of patients with subclinical TD, which was consistent with the previous findings of Pollack et al.[15]. The mechanism may be that the Th1/Th2 imbalance and proinflammatory state caused by high levels of adipokines (leptin, adiponectin, resistin, visfatin) and cytokines (eg, tumor necrosis factor-α, interleukin-6 and interleukin-1β) in patients with high BMI.

We found that the incidence of hypothyroidism and hyperthyroidism were 47.7% and 10.3% respectively in PLC patients treated with PD-1, hypothyroidism was 4.6 times higher than hyperthyroidism. Previous meta-analysis had reported that the incidence of hypothyroidism after PD-1 treatment was 10–16.4%, and the incidence of hyperthyroidism was 9–10.4%. There was little difference between the two, but our study showed that the incidence of hypothyroidism was much higher than that of hyperthyroidism. Previous meta-analysis [16] reported that the incidence of hypothyroidism after PD-1 treatment was 10–16.4%, and the incidence of hyperthyroidism was 9–10.4%. There was little difference between the two, but our study showed that the incidence of hypothyroidism was much higher than that of hyperthyroidism. The incidence of hypothyroidism in these patients is high, but not serious. Less than 1/3 of them have clinical symptoms, and only 7.5% of them have adverse reactions above grade 3. On the contrary, the incidence of hyperthyroidism in these patients is low, but more than 2/3 of them have clinical symptoms, and 15.0% of them have adverse reactions above grade 3. Among the 10 patients with severity above grade 3, there were 3 patients with hyperthyroidism and 7 patients with hypothyroidism. Except that 2 patients with hyperthyroidism did not fully recover and 2 patients with hypothyroidism died of liver failure, the remaining 1 patient with hyperthyroidism and 5 patients with hypothyroidism fully recovered after treatment. Therefore, as long as TD can be found in time and treated actively, most patients with TD have a good prognosis. The high incidence of subclinical TD suggests that we need to closely monitor thyroid function in the process of PD-1 treatment of PLC. When patients have common clinical symptoms of TD, such as fatigue, palpitation, loss of appetite, weight loss, edema and irritability, we need to be vigilant and give diagnosis and treatment in time to reduce the possibility of interrupting PD-1 treatment.

We also found some interesting differences. For example, the proportion of decompensated liver cirrhosis was higher in the hypothyroidism group, especially in the clinical hypothyroidism group and the primary TD group, and the proportion of patients with previous surgery was higher in the hyperthyroidism group, especially in the clinical hyperthyroidism group, and the proportion of patients with previous or combined use of targeted drugs was the highest in the primary TD group. Previous studies have found that the liver is an organ that has an important impact on thyroid hormone metabolism in addition to the thyroid [17, 18]. The transformation and inactivation of thyroid hormones require the participation of the liver, including metabolic processes such as deiodination, deamination and combined bile secretion, and the synthesis of thyroid hormone binding protein is also completed in the liver. If the damage of hepatocytes leads to the decline of liver function, the production of thyroid hormone and binding protein will be significantly affected, resulting in the decrease of thyroid hormone and even thyroid atrophy and degradation, especially in decompensated liver cirrhosis. Therefore, it is easier to develop hypothyroidism in patients with decompensated liver cirrhosis who use immunosuppressant such as PD-1 to produce TD. The reason why the proportion of patients with hyperthyroidism treated by surgical resection increased significantly may be related to the high probability of surgical resection caused by the early detection of PLC. Due to the high recurrence rate of liver cancer after operation, the total diagnosis and treatment time of these patients is longer because of the subsequent recurrence and treatment. Therefore, trauma and stress caused by surgical treatment itself, immune function damage, and the ability to receive more iodine containing contrast agents in the process of long-term diagnosis and treatment may be part of the inducement for hyperthyroidism after PD-1 treatment [19]. PD-1 combined with molecular targeted drugs was commonly used in the treatment of liver cancer. The targeted drugs were tyrosine kinase inhibitors (TKIs), including sorafenib, renvatinib, bevacizumab and regofinib. Previous studies had shown that the probability of TD after TKIs treatment was high. Koizumi et al. [20] found that there were 7 (14.0%), 26 (52.0%) and 5 (10.0%) patients with subclinical hypothyroidism, dominant hypothyroidism and thyrotoxicosis in 50 patients with advanced liver cancer treated with renvatinib. At present, it was considered that the mechanism of TD caused by TKIs drugs may be related to the effect of drugs on tyrosine kinase in thyroid vascular function. For example, TKIs inhibits blood vessels, leading to ischemic thyroiditis, causing transient thyrotoxicosis, or hypothyroidism due to the decrease of thyroid blood supply and the gradual destruction of thyroid gland [21, 22]. In addition, TKI could also affect the synthesis of thyroid hormone by inhibiting the transport of iodothyronine [23] and produce immunostimulatory properties by inhibiting the expression of CTLA4 and PD1 on CD4 + T and CD8 + T cells [24]. Therefore, PD-1 inhibitors combined with targeted drugs were more prone to primary TD in the treatment of liver cancer.

## Conclusion

To sum up, by analyzing the changes of thyroid function in PLC patients treated with PD-1 inhibitors in the real world, our study found that the incidence of TD was higher than that in previous clinical trials, but the degree of adverse reactions was mild. Patients with grade 3 adverse reactions could recover after treatment, and most of them did not need to interrupt PD-1 treatment. The clinical types of TD are mainly primary, subclinical and hypothyroidism. Some combined treatment schemes also have a certain impact on TD. In addition, since female were more likely to have thyroid diseases than male, the low proportion of female in this study may affect the research results. Therefore, it is necessary to detect the thyroid function of PLC patients using PD-1 inhibitor regularly and intervene in time to ensure the smooth progress of treatment.

## Data Availability

All data generated or analysed during this study are included in this article. The other raw datasets used and/or analysed in this study will be made available upon reasonable request to the corresponding author.
